# Addiction: Choice or Compulsion?

**DOI:** 10.3389/fpsyt.2013.00077

**Published:** 2013-08-07

**Authors:** Edmund Henden, Hans Olav Melberg, Ole Jørgen Røgeberg

**Affiliations:** ^1^Centre for the Study of Professions, Oslo and Akershus University College of Applied Sciences, Oslo, Norway; ^2^Department of Health Management and Health Economics, University of Oslo, Oslo, Norway; ^3^Ragnar Frisch Centre for Economic Research, Oslo, Norway

**Keywords:** addiction, compulsion, irresistible desires, choice, rationality

## Abstract

Normative thinking about addiction has traditionally been divided between, on the one hand, a medical model which sees addiction as a disease characterized by compulsive and relapsing drug use over which the addict has little or no control and, on the other, a moral model which sees addiction as a choice characterized by voluntary behavior under the control of the addict. Proponents of the former appeal to evidence showing that regular consumption of drugs causes persistent changes in the brain structures and functions known to be involved in the motivation of behavior. On this evidence, it is often concluded that becoming addicted involves a transition from voluntary, chosen drug use to non-voluntary compulsive drug use. Against this view, proponents of the moral model provide ample evidence that addictive drug use involves voluntary chosen behavior. In this article we argue that although they are right about something, both views are mistaken. We present a third model that neither rules out the view of addictive drug use as compulsive, nor that it involves voluntary chosen behavior.

## Introduction

The view of addiction as a neurobiological disease characterized by compulsive and relapsing drug use has come under renewed attack by several philosophers and psychologists (1–7).^1^ Their critique is partly empirical, partly conceptual. According to the empirical criticism, the disease view is not supported by the empirical evidence appealed to by its proponents. This includes biological evidence of changes to the normal operation of the brain caused by regular consumption of drugs as well as observational evidence of addicts’ repetitive self-destructive behavior. But this is insufficient, the critics claim, to warrant a conception of addiction as a disease. The biological evidence is of neurobiological correlates of drug use such as increased levels of the neurotransmitter dopamine, but these correlates are common to most forms of pleasurable experience (2, 3, 5). Sweet food, lottery prizes, sex, and exercise all create strong desires oriented toward some reward, and all essentially involve the same type of brain changes. There is nothing pathological about strong desires.

The second type of empirical evidence relates to the alleged compulsive patterns of self-destructive behavior often observed in addicts, and frequently accompanied by strong ambivalence: the addict expresses a desire not to consume drugs prior to, after, or even during the drug intake (8–11). The assumption is that this reveals the neurobiological effects of drug use to be significantly different from the seemingly similar effects of other desired activities or goods. Whereas strong desires ordinarily do not remove a person’s ability to control her behavior, addictive desires do, so the argument goes. Against this, the critics point out, there is plenty of evidence that addictive behavior involves voluntary, intentional, even rational actions. Indeed, under close scrutiny the drug-oriented behavior of addicts is shown to be less unusual than it may appear: it is influenced by a variety of incentives such as financial concerns, fear of arrest, values regarding parenthood, etc.; studies of addictions in the general population find moreover that most addicts quit drugs by their mid-30s, often without assistance (2, 4). How drug users describe loss of control depends variously on the appearance and characteristics of the person surveying them (12), and if we are to believe the experimental evidence it is *the believed* alcohol content rather than *the actual* alcohol content that influences how much alcoholics drink. Such evidence, it is argued, shows both that addicts can be persuaded to exercise their capacity for self-control if they are given what appear to them to be sufficiently good reasons, and that statements regarding loss of control are – at least to some extent – factually inaccurate and motivated by a desire to shift attribution of behavior from choices to circumstances.

Of course, nobody denies that addicts tend to pursue behaviors associated with risk and self-harm. But so do people who eat junk food, drive over the speed limit, have a sedentary lifestyle or practice base jumping. It seems excessive to argue that all such instances of risky, potentially harmful behavior are involuntary. The reason people often engage in such behavior is because they consider the benefits outweigh the costs. The ambivalence of addicts toward their addictive behavior is less typical of those pursing these other risky behaviors, but this too might have another explanation: given the stigma of addiction, proclaiming a desire to quit and helplessness in the face of “addiction” could be a functional device – something addicts just *say*, either because they are self-deceived or because they wish to defer responsibility for their socially unacceptable behavior (2). Some critics of addiction as disease believe that “addicts,” as we tend to think of them, do not really exist; there are only people who make bad decisions (7).

The conceptual critique of the disease view questions what it means to say that addiction is a “disease” that is characterized by “compulsive” and relapsing drug use. How can we reconcile a view of addictive behavior as a disease with a view of behavior-in-general as a choice? How do they differ and why? Clearly, one difference is that addiction involves behavior which in some sense is “out of control.” But in common usage, the notion of “out of control” ordinarily represents a continuum, ranging across cases of non-voluntary, non-intentional behavior, such as reflexive knee jerks, to cases of highly voluntary, intentional – even rational (in a welfare-maximizing sense) – actions involving self-harm due to ignorance or lack of foresight. Where along this continuum should addiction be placed?

Critics of the disease view assume that the notions of “disease” and “compulsion” commit its proponents to locating it closer to the non-voluntary end of the continuum, while the empirical evidence, they argue, in fact suggests a location closer to the rational end (2–4). They appear to have two main reasons for this contention. First, while symptoms of typical diseases such as Alzheimer’s or cancer are biologically based and non-voluntary in the sense that they do not develop as the result of decision-making processes but are beyond the person’s capacity to volitionally influence, this is not so in the case of the repetitive drug-oriented behavior of addicts. Although this behavior is the most prominent symptom of addiction, its development is clearly affected by decisions made and is volitionally influenced (4). It is flexible, adaptable, and involves elements of planning. Second, to claim that addictive behavior is compulsive means that it is caused by irresistible desires (2, 4–6). Irresistible desires, however, should not be affected by the presence of competing incentives. After all, the notion that “nothing else can compete” is a defining feature of irresistibility. Nevertheless, addictive behavior *is* clearly influenced by incentives.

In our opinion, these arguments show that a certain strong version of the disease view should be abandoned. However, we want to argue, another – weaker and more plausible – version of this view is still available. This view places addiction closer to the middle of the above-mentioned continuum. Our argument – and our article – has three parts. First, we argue that behavior can be *compulsive* even if it is not caused by *irresistible* desires. In support of this, we present evidence that demonstrates that behavior is commonly categorized as “compulsive” in clinical practice even if it is not caused by irresistible desires. Now, some critics of the disease view have taken the observation that addictive behavior is not caused by irresistible desires as a springboard to suggest that addictive behavior must in fact be ordinary rational behavior instead. The most systematic development of this view of addiction as ordinary rational behavior has taken place in the economics literature. In the second part of the article, we argue that the causal mechanism proposed by advocates of the so-called “rational addiction theories” in economics is both theoretically implausible and empirically false. Ambivalence is pervasive in addiction and irrationality appears to be the hallmark of addictive behavior. In the third part we return to the question of what constitutes addictive behavior as compulsive. If it is not the irresistible desire, what is it? We would like to present an alternative view, one based on a long tradition in philosophy and psychology. According to this view, addictive behavior is compulsive because it counterfactually depends on a motivational mechanism that systematically causes dissociation in the addict’s decision-making machinery. While the mechanism does not remove the addict’s ability to control her drug-oriented behavior, it sharply increases the effort she has to make to take advantage of alternatives to drugs compared to non-addicts. This view, which fits well with recent evidence in neuroscience, is not only consistent with the claim that addictive behavior is characterized by compulsive and relapsing drug use, it is also consistent with the claim that addiction involves voluntary, intentional behavior that is motivated by the addict’s decision-making processes.

## Addiction and Irresistible Desires

In a recently published book, Heyman argues that addiction is not a neurobiological disease because addictive behavior develops as the result of addicts’ decision-making processes and thus is within their capacity to volitionally influence (4). Although Heyman is clearly correct in saying that addiction involves voluntary behavior, that does not rule out a view of addiction as a mental disorder – which is its current medical diagnosis (13). This is worth mentioning, not least because few would claim that the symptoms of mental disorders necessarily develop independently of the persons’ decision-making processes and beyond their volitional influence. The whole point of psychological therapy depends on the ability in at least some of the victims of mental disorder to volitionally influence the symptoms of their disorder and learn how to exercise that capacity by attending psychological therapy. In other words, if addiction is a mental disorder, there is no obvious reason why we should be committed to the view that the symptoms of addiction – i.e., repetitive drug-oriented behavior – must be *non-voluntary* or unresponsive to incentives. Now, compulsion is clearly a symptom of mental disorder, but to what extent is it correct to view addictive behavior as compulsive? This, obviously, depends on how we define compulsive behavior.

The point of departure in most philosophical discussions of compulsive behavior is not diagnostic practice in psychiatry, but rather a concern with the metaphysics of free will. It has been commonly assumed that compulsive behavior involves a loss of freedom of the will.^2^ Many philosophers have therefore tended to conceptualize compulsive behavior as analogous to instances of interpersonal compulsion where someone is forced by someone else to act against her will. They have therefore tended to treat the notion of “compulsive behavior” as analytically equivalent to “compelled behavior” (2, 14–17). In the interpersonal case, the “compelling agent” is another person, while in the intrapersonal case it is an irresistible desire. A reasonable question is whether this conceptualization is consistent with the defining features of compulsive behavior used as diagnostic signposts in clinical practice.^3^ If it is not, metaphysical analysis will clearly be of little use for understanding real world behavior categorized as compulsive (as opposed to behaviors in philosophical thought-experiments). Clinical descriptions of compulsive behavior tend to emphasize a number of features (18–20): first, compulsive behavior is strongly cue-dependent in the sense that it is regularly triggered by certain situations, places, or people associated with the type of behavior in question. Second, compulsive persons feel repetitively driven to perform the behavior, often in spite of themselves; reports of feelings of compulsion are therefore common. Third, if compulsive persons sincerely try to refrain from acting upon their compulsive motivation, achieving success becomes, they report, increasingly difficult over time. These factors are present in obsessive-compulsive disorder (OCD) and impulse-control disorders (ICD) such as kleptomania (compulsive stealing), trichotillomania (compulsive hair-pulling), and compulsive buying. But is this kind of compulsive behavior consistent with the philosophical view that equates compulsiveness with irresistible desires?

In order to answer the question, we need first to be clear about what it means for a desire to be “irresistible.”^4^ A desire is irresistible at some time *t* to a person *S* if *S* is unable at *t* to resist acting on that desire. Put differently: if a person’s action was caused by an irresistible desire, it was *literally impossible* for her to *not* perform that action. However, there have been different accounts of precisely what kind of powerlessness this implies. Very generally, these accounts can be divided into two main groups. The first group covers what might be called “desire-centered accounts,” accounts which seek to explicate powerlessness in terms of the abnormal strength of the desire to perform the compulsive action. This desire is presumably so strong that no other motives can realistically compete. The second group, includes what might be called “control-centered accounts,” they seek to explicate powerlessness in terms of a loss of normal capacity for rational self-control. Let us quickly run through some examples of each of these account categories and see if they can adequately characterize the compulsive behaviors associated with clinical cases of compulsion.

Starting with the desire-centered accounts, they can be further divided into non-normative and normative versions. According to the non-normative desire-centered account, the compulsive person’s powerlessness is seen as an inability to resist acting on a desire because this desire, in virtue of its abnormal physical strength, is the immediate cause of the bodily movements made by the person in performing the compulsive behavior; no choice or decision has been made to make these movements. As one writer puts it, the appropriate interpersonal analogy to compulsion according to this view, is the thug who literally tosses me out of the room; just as I cannot help the way my body moves, so the compulsive person cannot help the way her body moves (21). On this account, compulsive behavior is non-voluntary and non-intentional. Compulsive people are powerless because they are physically incapable of refraining from their compulsive behavior. This account seems at odds with what is standardly referred to as compulsive behavior in the clinical literature. Compulsive behavior in clinical cases seems intentional and related to active choice. As one researcher remarks, in typical cases of OCD the person often carries out her compulsive behavior quite deliberately, taking particular care to carry it out precisely as she feels it ought to be done. If, for some reason, the behavior is disrupted, she will experience it as invalidated and in need of being restarted (19, 20). This suggests that the behavior can easily be delayed, reshaped, or substituted. Furthermore, there seems to be a growing consensus in the clinical literature that persons with OCD engage in their compulsive behavior in order to temporarily reduce the distress or anxiety associated with some obsession (18, 22). Usually they report a fear of disastrous consequences if the behavior is not properly carried out. There is clearly then an element of both purpose and control in OCD behavior that is at odds with seeing the behavior as non-voluntary and non-intentional in the sense implied by the non-normative, desire-centered account.

In contrast to the non-normative, desire-centered account, under the normative desire-centered account, the compulsive person’s powerlessness involves an inability to resist acting on a desire because the desire coerces her to *choose* the compulsive behavior (15, 23). This implies a normative notion of the strength of desire since what counts as “coercion” depends on what an individual can reasonably be expected to hold out against. The claim made by the normative desire-centered account is that the threat of unbearable psychological pain motivates the compulsive person in a way similar to how the threat of grievous bodily harm allows a robber to coerce his victims into handing over their money.

Though less starkly at odds with the clinical literature on compulsive behavior than the non-normative account, this account – too – fails to fit the facts. While compulsive persons clearly experiences distress in trying to refrain from compulsive behavior, there is little evidence to suggest that these feelings generally reach the level of “unbearable psychological pain.” Many researchers appear to believe, for example, that typical ICD-s, such as compulsive buying, are carried out to provide temporary relief or escape from feelings of general tension, whether it be depression, boredom, frustration, or some other negative mood state, often generating in the process certain soothing or pleasurable sensations (18). Whether preventing a person, on these occasions, from experiencing relief and pleasure – while no doubt unpleasant – is sufficient to create “unbearable psychological pain,” let alone a sense of “threat” similar to the robber’s threat of bodily harm, does not seem plausible.

A consideration that might provide further evidence against the coercion view is that compliance in cases of compulsion seems, in general, much harder to justify than compliance in cases of robbery (24). To see this, imagine we increased the cost of compliance in both cases: if you comply with “the threat” – i.e., hand over your money to the robber or use your money to buy something you don’t need in order to satisfy a compulsive desire – your child will go hungry for the next 2 weeks since you won’t be able to buy her enough food. If the coercive threats in these two cases were no different, we would not expect to see any difference in their respective justification of compliance. Arguably, though, there is such a difference. While it seems likely that most people would find it unreasonable to expect non-compliance on the part of the victim to the demands of the robber, it seems much less likely that they would find it equally unreasonable to expect non-compliance on the part of the compulsive buyer. If this is correct, people’s reactive attitudes to the victims of robbers would appear to differ in important respects from their reactive attitudes to compulsive buyers.

Even if the desire-centered accounts fail to characterize what is standardly referred to as compulsive behavior in the clinical literature, what about the control-centered accounts? Whereas the desire-centered accounts see compulsion as explained by the overwhelming force of drug-oriented desires, the control-centered accounts see it explained by a loss of normal capacities for rational self-control (14, 17). The difference between the two accounts is this: the former does not necessarily imply that compulsive people have lost their normal capacity for rational self-control. Thus, someone who fails to resist acting on some desire because of its abnormal physical strength doesn’t necessarily fail because she has lost her normal capacity to rational control herself – she may both possess this capacity and exercise it properly. The reason she fails is that she is overpowered by the superior force of her compulsive desire. Compare this with a case in which you are being tossed out a room by a thug: that you are being tossed out against your will doesn’t necessarily mean there is anything wrong with your will, e.g., that you have lost this capacity altogether, or that you have failed to exercise it properly. It might simply mean that you are overpowered by your opponent’s superior physical strength (21).

Depending on what is meant by “normal capacity for rational self-control,” there might be different views of precisely what kind of powerlessness the control-centered accounts implicate. Perhaps the most common is a reasons-based view, according to which rational self-control is understood in terms of reasons-responsiveness, and its loss as a lack of susceptibility to countervailing reasons (25–27). That is, someone has lost her capacity at some time *t* if a certain sort of counterfactual is true of her: if she were presented with what she took to be good and sufficient reasons for not performing some compulsive action at *t*, she would still perform that action at *t*. Given standard interpretation of the truth-conditions of such counterfactuals, it would be literally impossible for her to successfully resist, no matter what sort of incentives she is presented with. Does this account succeed in characterizing what in the clinical literature is standardly referred to as compulsive behavior?

Once again, there seems to be little reason to assume that it does. Supportive evidence can be gleaned from the apparent success of response prevention therapy as a treatment modality for OCD. The aim of response prevention therapy is to break the relationship between the various trigger situations which provoke the compulsive urge, and the compulsive behavior, by repeatedly exposing the compulsive person to different trigger situations but encouraging her to refrain from the compulsive behavior (20). For example, a compulsive person whose washing rituals are a result of an obsession about being contaminated by dogs, may be instructed to pat a dog and then refrain from washing her hands or to take a bath only after a given period of time. As the sessions are repeated, the interval is extended. Research shows that if the pattern is followed in each of the trigger situations, the cumulative effect is progressively less discomfort and desire to engage in the compulsive behavior. On the assumption that compulsive behavior involves a complete loss of the normal capacity for rational self-control, it is difficult to see how compulsive persons could successfully engage in this kind of exposure therapy. Without that capacity, how could they possibly comply with instructions to delay their response to a trigger situation? Inference to the best explanation suggests that they retain their capacities. What response prevention therapy does is to give them an incentive to put more effort into exercising them.^5^

To sum up. If the arguments of this section are correct, the term “compulsive behavior” as used by the critics of the disease view is not co-extensional with the term “compulsive behavior” as it is standardly used in the clinical literature. There is no implicit or explicit assumption in the clinical literature that compulsive behavior must be caused by “irresistible desires.” The term “compulsive behavior” simply refers to repetitive behavioral patterns performed in characteristic circumstances which the compulsive person finds it difficult to override by intentional effort. On this description, compulsive persons are not necessarily powerless with respect to their compulsive behavior. Neither does it rule out the possibility of this behavior being voluntary, intentional, and even motivated by the compulsive person’s decision-making processes. This does not, of course, show that *addictive* behavior is compulsive. Some critics of the disease view who argue that addictive behavior is not caused by irresistible desires appear to take this to suggest that addictions must involve ordinary rational behavior instead. As we noted above, the latter view has perhaps been most systematically developed in the economics literature, where so-called “rational addiction theories” provide the dominant model of addiction. In the next section, we argue that these theories fail to explain what is distinctive about addiction.

## The Theory of Rational Addiction

Viewing addiction merely as a specific pattern of rational choices obviates the need for a theory of addiction. Addictive behavior is nothing more than ordinary behavior, and needs no additional explanation. On the other hand, insofar as addictions typically involve a small set of substances and activities, these substances and activities must have something in common that makes an addictive form of behavior all the more likely. Then again, not everyone becomes an addict, and systematic differences have been found between high and low risk individuals. Such regularities require an explanation: if addictions are constituted by ordinary rational choices, why do they appear to be different from ordinary behavior?

One of the most extensively developed attempts to answer this question was proposed in the theory of rational addiction and its descendants [e.g., (28–30)]. According to this family of theories, the peculiar features of addictive behavior derive from the peculiar incentives that can arise when a good has lagged effects. It means in short that consumption of a good is consumption-for-enjoyment-now, but also crucially, an investment decision accommodating the lagged effects the individual expects to enjoy in the future. Given a particular pattern of short- and long-term effects, such as those produced by a drug, some consumers find that – for them – the best consumption plan is gradually to increase consumption to ever higher levels. These consumers display what the theory labels “addictions.” On this view addictive behavior is neither paradoxical nor troublesome. Drug users may be unhappy, but “they would be even more unhappy if they were prevented from consuming the addictive goods” (28). Differences between people are due to differences in time preferences, available choice sets, or uncertainty: if drug effects differ across consumers and individuals are uncertain about which effects they should anticipate, they will make a rational choice under uncertainty which, in the event, may turn out to be the wrong one, creating a situation where high-level drug use becomes the optimal way forward (30). Provided the behavior does not harm others, as Becker and Murphy (28) write, there is no reason to intervene. An addiction as far as the economic approach is concerned is simply an unproblematic matter of choice.

This conception of addiction as rational choice must be clearly distinguished from the view of rational individuals dealing with addictions. A rational choice proponent need not necessarily see addictive behavior as rational. They could view addiction as a disease that hijacked our decision-making apparatus and reduced our autonomy, so that our rationality would be reduced *given* an addiction. However, rational choice would still be involved *before* the addiction took hold: the risk of “getting addicted,” the rational choice proponent could argue, is a rational choice for some, involving a trade-off between the risk of losing autonomy on the one hand, and something else that is valued on the other – pleasure, respite from boredom, etc. This, however, would not be a theory of addiction as such, since it would not explain how or to what extent addiction reduced the individual’s autonomy.

The theory of rational addiction, by contrast, is a *theory of addiction*. To quote Becker and Murphy again, their claim is that “rational choice theory can explain a wide variety of addictive behavior.” In addition to intoxicants and cigarettes, they mention addictions to “work, eating, music, television, their standard of living, other people, religion, and many other activities.” Their explanation of such behavior requires goods or activities to have lagged (i.e., delayed) effects on the user. Addictive goods can be both beneficial and harmful. Harmful addictions, which are the most interesting, require that the good or activity has two properties. First, current consumption reduces your future “baseline” welfare. A high sugar intake makes you fatter tomorrow, which you may dislike. Smoking today makes you cough up phlegm the next morning and feel a bothersome appetite for more cigarettes. Second, current consumption increases the future value of a unit of the addictive good itself. If your growing waistline makes you sad, it gives “comfort food” more sadness to work on. As your body’s craving for nicotine increases, cigarettes can help satisfy this renewed urge in addition to giving you the benefits it already did. As a rational individual, you balance these effects and work out the plan for future consumption that would maximize your welfare.

An important but often ignored criticism of the theory of rational addiction as a theory of how people actually come to be addicted points to the incredible amount of intricacy all this planning involves. In deciding whether to smoke a cigarette here and now, you are actually designing and evaluating a plan on cigarette-smoking-starting-now-and-far-into-the-future. You take into account expected changes in smoking legislation, tobacco taxes, the way current use affects tomorrow’s tastes, uncertainty regarding risks, etc. In this sense, the theory of rational addiction is much more elaborate than simply positing that individuals respond to incentives or rationally take into account the possibility of getting an “addiction-disease.” Instead, according to adherents, addictive behavior can usefully be viewed as a highly intricate and sophisticated plan that optimally solves a complex decision problem featuring delayed effects and uncertainty. Whether we find this to be useful will depend essentially on what we want to use the theory for.

## Evaluating the Theory of Rational Addiction^6^

The above description of rational addiction theory may make it sound “unrealistic,” especially to non-economists. However, as Mäki (31) warns, “[m]uch of the criticism of economics […] is based on the mistaken belief that criticism is easy – such as when inferring from unrealistic assumptions to models being incorrect […] it is not easy to reliably identify [the] flaws (of economics) almost regardless of how serious they are.” The reason is that economic models can serve a multitude of different aims, and the criteria against which they should be judged – and the evidence relevant to judging them – will vary with the aim.

The reason we need to point this out is because economic models can be insulated from criticism by claiming they are merely explorations of formal frameworks or false-but-useful ways of summarizing stylized facts. When we discuss rational addiction theory, however, we are interested in the theory as an attempt to explain the underlying causal mechanisms which generate addictive behavior in the real world. This view of the theory is common both among contributors to the written literature and researchers working on them: a survey of researchers with peer-reviewed publications on rational addiction theory found 39% of them agreeing that the rational addiction literature “provides insights into how addicts choose that are relevant for psychologists and treatment professionals,” while 56% agreed that the literature “contains insights on the welfare consequences of addictive goods and public policies toward these” (32).

This, in our view, is mistaken [see (33, 34) for details and supporting references]. The shortcomings of the theory in this respect may generalize to other attempts at explaining addictions as ordinary and rational behavior. At a broad level, the problem is that addictions are characterized by seemingly flip-flopping attitudes and ambivalence, self-control issues, regret, etc. The addicts fail to verbalize motives for their actions that would make them understandable, sensible, and time-consistent, and in the absence of simple and recognizable motives for the behavior, the rational choice believer is compelled to posit ever-subtler, sophisticated but ultimately non-credible motives and incentives to explain the behavior. Rational addiction theory and its variants exemplify this problem. They explain addictive behavior patterns as the result of optimal choices given a specific choice problem. The shape of the optimal consumption path is determined by the structure and strength of the lagged and immediate effects of the good, as well as the consumer’s time preferences and other consumption opportunities. By varying these and the number of lagged effects we can generate a variety of consumption paths: rising, falling, cyclic, chaotic. In other words, the optimal consumption plan is sensitive to details in the choice problem facing the consumer. Empirically, it is hard to identify the actual decision problem facing any particular individual. The incentives consist of the “net” subjective valuation of a bundle of different effects on health, psyche, etc. In this sense, the theory “explains” behavior in terms of a detailed hypothesis about unobservable and non-measurable mental constructs. We could identify the lagged “effects” of the drugs with more objectively measurable effects, such as those on health, but this will not solve the problem. The actual lag structures of harmful effects are unlikely to match the assumptions that generate “typical” addictive patterns, and the effects on disease risk, the body and future tastes are not known to the required level of precision in the scientific literature. Since real addicts are supposed to face and have solved this decision problem, it is a further problem for the theory that surveyed individuals state beliefs about lagged effects that are in clear contradiction with the required assumptions, and it is a further problem that even educated experimental subjects generally fail to find the optimal solution to structurally identical investment problems in experiments where this could earn them actual money (35). Put differently: the choice problem that is claimed to generate addictive behaviors is neither the one individuals actually face, nor the one they believe themselves to be facing, nor indeed the one the average smoker or “junkie” is likely to recognize if it was explained to them, nor a choice problem they would be able to find the optimal solution to in practice. The “typical” addictive consumption pattern is more stable over time and across people than it “should be” according to the theory. The cross-sectional variation in beliefs about effects of drugs and the time-variation in knowledge about the effects of drugs should generate a variety of drug use patterns rather than the ones taken as a stylized fact by the theory. The theory says that factors X generate Y, but there is no evidence that X is present where Y is present, and no evidence that experimental manipulations that create factors X lead to behavior pattern Y. The theory, quite simply, is not credible as a description of the underlying choice processes generating addictions, and there are no plausible arguments showing why selection effects or simpler heuristics would allow most people to search for and implement these optimal paths in alternative ways.^7^

That addiction involves ordinary rational behavior is not a tenable proposition in our view. On the contrary, we believe there are good reasons to assume that addictive behavior is compulsive in the clinical sense of the DSM-IV. Yet what DSM-IV gives us is, of course, a purely descriptive sense of “compulsive.” It does not explain what makes these behavioral patterns compulsive. If their compulsivity is not constituted by an irresistible desire for drugs, what does constitute them? And what kind of evidence would show that addictive behavior *is* compulsive? The key to answering these questions lies neither in the abnormal strength of addictive desires, we contend, nor in a loss of normal capacities for rational self-control, but rather in certain special features intrinsic to addicts’ decision-making processes.

## Addiction and Compulsive Behavior

Compulsion, somewhat paradoxically, seems to involve deliberate, goal-directed behavior caused by something that is external to the person and independent of practical deliberation. On the one hand, something seems to assail the person – as if from without. On the other hand, it is an essentially active phenomenon, a kind of intentional behavior aimed at altering mood states or regulating affect. There is, in other words, an appearance of control in a class of behaviors largely defined by loss of control. We believe that both these features need to be addressed by our notion of compulsion. Philosophers, often motivated by a concern with the metaphysics of free will, have focused exclusively on the apparent loss of control. However, their picture of compulsive behavior as motivationally compelled, and of addictive behavior as compulsive due to the *irresistibility* of its psychological antecedents, is difficult to reconcile with the intentionality and controlled nature of such actions and with how the term “compulsive behavior” is applied to clinical cases. Instead, we want to suggest that compulsive behavior can be understood in terms of persistent patterns of failed decision-making caused by a dissociation in the person’s volitional control. This analysis does not entail that the person must have lost her capacity to resist. We also believe it fits better with the application of this notion to clinical cases. If we are on the right track, then there is good reason to say that one of the defining features of addiction is precisely its compulsive nature.

It has been common in much philosophy to treat volitional control as more or less the same as rational self-control, that is, a capacity persons have to bring their actions into line with what they judge to have most reason to do. Persons exercise rational self-control by directing (in various ways) their attention away from rebellious desires in order to form, retain, or execute intentions to do what they consider to be the most valuable course of action. On the assumption that all there is to volitional control is rational self-control, it follows that a failure of rational self-control necessarily is a failure of volitional control. This has the implication that when persons’ behavior is “out of control,” it can only be due to some force over which they have no control, a motivational element that is completely external to their volitional capacities, e.g., a desire that causes their behavior directly, independently of their decision-making system.

This conflation of volitional control and rational self-control makes it hard to handle even standard cases of compulsive behavior. Compulsive persons sometimes make conscious and active efforts to do *the opposite* of what they take themselves to have most reason to do, in some cases by using attention-managing strategies to block out thoughts about these reasons (36).^8^ Consider, for example, the sort of ritualistic compulsions typical of OCD. Persons with OCD tend not to want to be interrupted or distracted while engaged in these rituals. As we have seen, the clinical literature indicates that they serve a distinct function, namely to reduce negative feelings. Yet there is no logical connection between the description under which they intentionally engage in behavior of this sort (say, checking, hoarding, or washing) and the goal to which their actions are directed. So while they may make an active decision and spend considerable effort translating that decision into action, the behavioral pattern itself seems recruited through some associative or implicit learning process independent of practical deliberation and voluntary control. Consequently compulsive persons often regard their own action as excessive, unpleasant, and pointless. That is, they do not associate anything pleasurable or desirable with its performance. It does not seem plausible that they are driven by a strong cognitive desire to perform it. Nevertheless, they perform it intentionally. They even make an effort to do it “properly.” Initiating and executing compulsive behavior of this sort clearly require considerable amounts of volitional control, even though it may involve a failure to pursue what the compulsive persons themselves consider the most valuable course of action.^9^ Rather than being characterized by *a loss of volitional control*, therefore, we believe it is more plausible to say that compulsive behavior is characterized by *a dissociation in volitional control*. This view finds further support in the observation that compulsive persons often appear to recognize at the same time as they are deciding to perform the compulsive action that it is a mistake (“It is a pointless to wash my hands yet again”), yet go on to intentionally form and implement their decision to undertake that action in spite of these normative considerations. In fact, dissociative experiences, such as feelings of “standing outside oneself while acting,” are frequently reported across a range of compulsive phenomena, including addiction to drugs (37). There seems, in other words, to be a disparity between compulsive persons’ decisions and actions on the one hand, and their evaluative preferences (what course of action they judge most valuable) on the other, which can be explained neither in terms of a failure of volitional control nor in terms of the abnormal strength of their cognitive desire to engage in the behavior.^10^

We believe the possibility of this kind of failed decision-making – the irrationality of which is displayed in a form of incoherence in the person’s attitudes – is an essential feature of compulsive behavior. One way of understanding this phenomenon is by distinguishing between two ways of making choices. Postulating the existence of a duality behind people’s choices is, of course, not new. It has a long history in philosophy and psychology, reaching back to Aristotle’s distinction between the rational and non-rational part of the soul.^11^ While most early work on this duality was conceptual in nature and based on informal observations of human behavior and personal introspection, the development of what have become known as “dual-process theories” only started with the cognitive revolution in psychology in the 1960s and 1970s. Experimental studies of attention, learning, memory, and reasoning were important influences (38). Since then, a wide variety of evidence has converged on the conclusion that some sort of dual-process notion is needed to explain how the overall process of decision-making occurs (39, 40). Unlike before, we are now beginning to understand the biology and cognitive structure of the different parts (41). According to dual-process theory, decision-making can broadly be divided into two modes, one fast, intuitive, and effortless shaped by biology and implicit learning, the other slow, analytical, and effortful shaped by culture and formal tuition. While the former mode – in the dual-process literature often referred to as type-1 processes – depends on environmental cues, is associative, automatic, and can control behavior directly without need for controlled attention, the latter – often referred to as type-2 processes – depends on de-contextualization, is rule-based and requires controlled attention and effort. There is much disagreement about precisely how these processes should be characterized and distinguished (39, 42). We cannot enter into this debate here. For present purposes, what matters is that dual-process theory allows a closer scrutiny of the vulnerable aspects of the decision-making process by permitting focus on the important ways in which the different modes of decision-making interact.

To achieve rational decision-making the two modes have to work well together to reliably contribute to the person’s goal achievement (43). This requires two things. First, that the person’s type-2 process can exert an executive function and override the impulsive output of her type-1 process. For this to happen, her type-2 process must be able to generate a more considered response that is in line with her normative reasons, as well as involve inhibitory mechanisms to suppress the response tendencies of her type-1 process. Second, it requires that the person’s type-1 process selects adequate and relevant information about the practical situation that can provide input for her type-2 process (41). There is widespread agreement that some form of automatic, pre-conscious processing determines the person’s locus of attention and what stored memories and beliefs are recalled for as relevant to her current situation. This suggests that the person’s normative reasons (represented in her type-2 process) might be shaped, even to a significant degree, by type-1 encoding of information about which aspects of the situation need to be taken into account, and which can be ignored.^12^

Given this view of rational decision-making, *failed* decision-making can be seen to arise in two ways. One might be if the decision-making process of either type is internally biased. This could be the result of either an under or over appreciation of certain contextual cues, a failure to ignore distracting features, or a process for combining and processing information that uses simplified algorithms with potential biases. If a type-1 process fails to encode relevant information, or encodes irrelevant information that enters into the person’s subsequent analytic type-2 process, it may cause blindness to special circumstances or to longer-range goals. In consequence the person’s flexibility to be able to consider alternative reasons for acting is systematically undermined. Decision-making can also fail if a conflict arises between type-1 and type-2 processes. In the standard scenario, the person fails to suppress an intuitive but non-normative response generated by her type-1 process despite the fact that it conflicts with a considered normative response generated by her type-2 process. In contrast with cases of internally biased decision-making, the possibility of such executive failures implies that the person may be consciously aware while making her decision that it is mistaken. There seems to be no clear reason to assume that executive failures of this sort must presuppose that the person has any abnormally strong cognitive desire to respond in line with her type-1 process or has lost her ability to refrain from responding in this way. Rather, the failure is simply one of putting insufficient effort into overriding a type-1 process. That such failures occasionally occur should not surprise us given the cue-dependency, computational speed, and dissociated nature of type-1 processes. These decision processes plug more or less directly into the person’s motor system and seem almost to have “actional” character. Type-2 processes, on the other hand, require an effort that tires the person and may make it harder to engage in type-2 reasoning and override type-1 processes resulting in so-called “decision-fatigue” (5, 44).

We believe it is plausible that compulsive behavior involves persistent patterns of failed decision-making in one or the other of these two senses.^13^ In the former, a mis-contextualization of information enters into the person’s subsequent analytic type-2 process and causes a biased response, and in the latter, there is an executive failure to appropriately engage type-2 processes or use their output to override the impulsive output of a type-1 process. In either case, when failed decision-making *occurs repetitively, leading to a maladaptive behavioral pattern* deserving of the label “compulsive,” it is plausibly because some type-1 process has become fixed in inert dispositions and patterns of perception and response. Presumably, there is some underlying cue-triggered motivational mechanism which – perhaps due to repetition and reinforcement – has become deeply entrenched. In cases where there is subjective experience of conflict, this mechanism repeatedly pulls the person’s decisions away from her evaluative preferences. In cases where there is no subjective experience of conflict, she may be led to consistently ignore special circumstances or longer-range goals and hence systematically undermine her ability to consider alternative reasons for acting.^14^ Whether or not there is an experience of conflict in a particular case may be a matter of the psychological resources of the individual (e.g., insight vs. tendency to rationalize or confabulate reasons for actions generated by her type-1 process). In either case, the persons’ maladaptive behavioral patterns may counterfactually depend on the same type of entrenched motivational mechanism.

If this general picture of compulsivity is on the right track, is there any reason to believe that *addiction* involves compulsive behavior?^15^ The best reason is that addictive behavior appears to be strongly cue-dependent (9, 45), and that addicts regularly and systematically fail to take advantage of alternatives to drugs in spite of negative consequences for themselves, and often in spite of what they judge to be the most valuable course of action (11). There is a tendency for addicts to systematically ignore or downplay the costs of taking drugs while greatly exaggerating its benefits (4). As George Ainslie has argued, addicts often frame their decisions temporally as repeated, independent choices between alternatives one at a time on the basis of their immediate costs and benefits, rather than as single choices over series of similar alternatives across time on the basis of their summed costs and benefits (46). In such cases the addict’s decision-making process is clearly biased since she concentrates too much on the immediate benefits of drugs and systematically ignores her longer-range goals. From a conceptual point of view, what could explain such biasing within a dual-process approach is that a drug-oriented type-1 process – due to the entrenchment of some underlying cue-triggered motivational mechanism – has become fixed in a pattern of perception and response that systematically fails to encode logically relevant information about drug-related situations. When such drug-oriented type-1 processes regularly shape the content and locus of attention of a person’s subsequent analytic type-2 processes, they cause the rate at which she discounts the value of future rewards, such as, e.g., the benefits of abstinence, to increase drastically relative to the rate at which she discounts consumption. The result is that when opportunities for consumption arise, the person’s estimate of their values has increased so much more relative to her estimate of the value of abstinence that her preference reverses. But in addition to such cases of so-called “hyperbolic discounting” we believe (more controversially) that there is also evidence of cases in which addicts fail to take advantage of alternatives to drugs in spite of judging, at the moment of choice, that the value of abstaining is higher than the value of consumption. In these cases the problem is not internal biasing of analytic type-2 processes, but rather a regular failure to override type-1 processes which systematically fail to encode relevant information about drug-related situations.

Evidence of such dual-process conflicts in addiction comes from the observation that many addicts appear to make conscious and strenuous efforts to exercise restraint at the same time as they are seeking or actually taking drugs. As addiction researcher Robert West puts it: “When the restraint fails, there is often (but not always) no sense of the addict having changed his mind and deciding to engage in the behavior as a positive step; rather the sense is of a failure to exert control followed by regret and a feeling of having let oneself down” (47). As neuroscientists Kent Berridge and Terry E. Robinson note in a similar vein, there is plenty of evidence that addicts often continue to seek and take drugs even when no pleasure can be obtained, even in the absence of withdrawal – even – in fact when they are convinced that taking drugs is a disastrous course of action for them (11). Addicts, this would suggest, will often continue to judge that abstaining is the most valuable course of action even as they are carrying out their drug-oriented behavior.

What explains these kinds of systematic errors in drug-oriented decision-making? Specifying the nature of the underlying mechanism(s) is an important component of the empirical explanation of the compulsive character of addictive behavior. Our aim in this article has not been to propose any such explanation, but to suggest how to make conceptually sense of the “compulsive character” of behavior in a way that does not depend on the notion of irresistible desire and, in addition, to provide evidence in support of the view that addictive behavior *is* compulsive in this sense. That being said, an example of a mechanism that seems to fit well with the dual-process analysis of compulsivity is that proposed by Berridge and Robinson in their influential work on incentive-sensitization. Incentive-sensitization is a mechanism whereby repeated drug use produces a dopaminergic response that becomes sensitized by causing certain regions in the brain involved in the motivation of behavior to be more easily activated by drugs or drug-related cues independent of the addict’s cognitive desires, judgments, or “likings.” In a series of papers Berridge and Robinson provide evidence that the psychological process and neural substrate responsible for determining cognitive-affective liking are separable from the psychological process and neural substrate responsible for determining incentive salience – the degree to which a goal or stimulus is action-driving – or what they call “wanting” (48). While normally “liking” and “wanting” go together so that we “want” the things we “like” (e.g., the hedonic value associated with some environmental cue or circumstance serves as a trigger to activate and direct “wanting”), in addiction they come apart, making addicts “want” things they do not “like.” The reason incentive-sensitization might give us an empirical explanation of why addictive behavior is compulsive in the sense we have characterized compulsivity in this article is that it might explain why addicts’ drug-oriented type-1 processes become fixed in inert dispositions and patterns of perception and response which lead to systematically biasing of their type-2 processes or to the creation of regular conflicts between their type-1 and type-2 processes. Continuous failures to override type-1 processes that are dependent on an entrenched motivational mechanism like incentive-sensitization arise since these processes are difficult to override by intentional effort due to their cue-dependency, computational speed and frequency, and because addicts simply put insufficient effort into overriding them (perhaps due to decision-fatigue, misjudgment, or some other reason).^16^ This, of course, is perfectly consistent with the common observation that quitting drugs is hard if you are an addict, but without entailing that this is because addicts are driven by irresistible desires for drugs or have lost their powers of resistance. A dual-process analysis of the notion of compulsivity does not, therefore, rule out the intentionality of addictive behavior. However, nor does it rule out the possibility that some other psychological or neurological mechanism (or combination of mechanisms) than incentive-sensitization might in the end turn out to provide the best empirical explanation of the compulsive behavior of human addicts (or at least be part of such an explanation).^17^ Ultimately, it is a matter for the sciences to decide the precise nature of the relevant mechanism(s) which create(s) persistent dissociations in addicts’ decision-making machinery – or indeed of any of the various mechanisms that might be responsible for the different compulsive disorders.

## Conclusion

The question posed in the title of this article would seem to necessitate an either/or answer: *either* addiction involves voluntary, chosen behavior and is therefore not compulsive *or* it involves compulsion and therefore is not voluntary, chosen behavior. The bone of contention over which the respective proponents of the medical and the moral model of addiction do battle seems to rely in large part on the assumed contradiction between these two answers. The normative implications are obviously deep and far-reaching. If addiction rules out voluntary behavior and choice, then addicts can only (at best) be indirectly responsible for their drug use. That widens the scope for public policy interventions. If, by contrast, addiction involves voluntary, chosen behavior, this scope for intervention will be correspondingly constrained. Our aim in this article has been to argue that a middle path is not only possible but actually quite plausible in the light of the evidence: behavior can be voluntary, chosen, and compulsive at the same time. One way of making conceptual sense of this is to assume that our decision-making system is divisible. If such divisions stabilize due to the entrenchment of some underlying motivational mechanism and cause regular and systematic failures in the person’s decision-making with respect to actions of a certain type, they create compulsive behavioral patterns that may be very difficult for her to override by intentional effort alone. There are many good reasons, in our opinion, to believe that addictions essentially depend on such divisions in the decision-making system of addicts. However, this view does not mean that it is *literally impossible* for addicts to refrain from drugs. It only means it is much harder for them than it is for people who are not addicted. Even heavily addicted individuals have the capacity to abstain, although they may need help to learn how to exercise that capacity properly. We believe this mix of the “moral” and the “medical” model of addiction may open up for a more nuanced approach to many of the pressing normative issues raised by public policies, practices, and treatments in the addiction field.

## Conflict of Interest Statement

The authors declare that the research was conducted in the absence of any commercial or financial relationships that could be construed as a potential conflict of interest.

## Footnotes

^1^The disease view of addiction was also criticized as part of the earlier “anti-psychiatry” movement in the seventies [e.g., (49)], though this criticism seems to have had less impact on the general view of addictions in psychiatry and psychology.

^2^The notion of “free will” is, of course, notoriously difficult, and there is little agreement on how it should be analyzed. For present purposes, we assume that a person has free will with respect to a particular action at some time if she has the ability to refrain from that action at that time.

^3^In psychiatry the term “compulsion” is, of course, used to refer to a kind of symptom of many different disorders. When we use the terms “compulsion” and “compulsive” in what follows, what we have in mind will be the sort of repetitive physical behavior performed in characteristic circumstances that is typically referred to as “compulsive” in the clinical literature. We do not mean to suggest that compulsive behavior in this sense is a symptom of *all* compulsive disorders, nor that there is one specific mechanism that explains all observed varieties of compulsive behavior.

^4^By “desire” we mean in what follows *cognitive* desire, i.e., a motivating state that involves some favorable representation of the state of affairs that one acts in a certain way (e.g., as desirable, pleasurable, enjoyable, or good).

^5^It is worth noting here that not all versions of the control-centered accounts of compulsivity need be vulnerable to this kind of objection. The version we criticize is the one that equates compulsivity with a failure of what Fisher and Ravizza refer to as “strong reasons-responsiveness” (50).

^6^The following discussion is largely based on Rogeberg and Melberg (34), to which we refer readers who want additional details.

^7^In our view the most interesting work on addiction from economists comes from researchers who try to establish a middle ground between viewing addiction as a disease that completely removes the addict’s ability to control his behavior and viewing it as nothing more than an extremely subtle and sophisticated form of maneuvering undertaken by the addict in the face of complex incentives. Bernheim and Rangel [(51, 52), see also (53)] have developed a theory, for instance, in which addiction is the outcome of processes that affect our expectation of pleasure or welfare derived from using the drug, as well as affecting the likelihood that we will make the consumption decision using a “cold,” rational decision process. Their work can be seen as an attempt to examine how rational agents would deal with addictions when the addiction itself is a disturbance of the choice process.

^8^This is, of course, not true of all cases of compulsive behavior. Often compulsive behavior becomes over-learned and automatized as a result of repetition and may require virtually no active efforts at all (54).

^9^We can specify the motivational economy in volitional vs. valuational conflicts by applying the four-type utility framework proposed by Kahneman and colleagues [see (55)]. In utility-maximizing terms, “volitional” could then be specified in terms of “decision utility,” and “valuational” in terms of “predicted utility.” See Berridge and Aldridge (56) for a practical illustration of how this framework can be used to explain irrational decision-making. Having said that, our general approach does not depend, we believe, on any specific view of the motivational economy involved.

^10^Although this conflicts with standard assumptions in economics, there are economic models of behavior that break the link between “optimal behavior” and “actual behavior” by including preferences or psychological quirks that are inconsistent with a pure rational choice model [see e.g., (51)]. In a pure rational choice model, the chosen action will always be optimal (or optimal by expectation), which “allows” the researcher to infer the welfare-determining preferences from the behavior (i.e., revealed preferences). With this link broken, the researcher has to provide a higher level of evidential support for the theoretical assumptions themselves, while welfare analyses need to distinguish between different concepts of welfare or “utility,” for instance along the lines of Kahneman et al. (55).

^11^“For in the continent and the incontinent person we praise their reason, i.e., the [part] of the soul that has reason, because it exhorts them correctly and toward what is best; but they evidently also have in them some other [part] that is by nature something besides reason, conflicting and struggling with reason.” (57), 1102b 15–20.

^12^A difficult philosophical issue concerns the account of the self and its rationality in face of the conflict described by dual-process theory. According to one influential view, we should identify the self with the “better” type-2 processes because only on this level do we notice and are bothered by a lack of rational integration (58). While we cannot address all the issues raised by this claim at this juncture [but see (43) for a discussion], for present purposes, we assume that in (at least) standard cases, the self is associated with type-2 processing.

^13^One important implication of a dual-process approach to decision-making is that each one of us is potentially vulnerable to developing compulsive behavioral patterns, including addictions. Redish and colleagues propose a variant of this approach as a unified framework for addiction where they distinguish between 10 different failure points in our decision-making system, each of which may be capable of driving a person to make addictive decision “mistakes.” The reason some people go on to develop addictions while others do not depends on the specific “interaction between the genetics of the individual, the developmental environment (social and physical), the developmental stage of the individual, and the behavioral experience with the addictive substance” [(59), 430].

^14^In the social cognition literature, one variant of this phenomenon is referred to as “cognitive narrowing,” which is a tendency to focus attention on an immediate, concrete, low-level task (e.g., shopping or buying) that prevents consideration of the longer term consequences of an action (36).

^15^One interesting issue concerns how this picture of compulsivity relates to the defects in action and agency discussed in the philosophical literature as “weakness of will” [see e.g., (60)]. Although we cannot address this issue here (the relevant literature is voluminous), one of the authors has argued elsewhere that there are some important differences between addiction/compulsion and weakness of will. See Henden (61).

^16^It is worth noting that this view of compulsivity, in relying on the notion of “level of effort” rather than “motivational irresistibility,” implies that compulsivity is a matter of degree. It is consistent, therefore, with the possibility of differences between forms of compulsive behavior in terms of how difficult they are to resist, e.g., between the kinds of behavior seen in, say, OCD and in the different addictions. For more on the conceptual connection between effort expenditure and compulsivity, see Henden (61).

^17^The incentive-sensitization theory of addiction remains controversial. Most of the data supporting it come from laboratory animal studies with little consideration of the social context in which the drugs were administered. This has caused some to criticize the theory for failing to generalize to human addicts [for a recent discussion, see (62)]. There is also evidence suggesting that incentive-sensitization by itself is not sufficient to create repetitive drug-oriented behavior in animals if they are given more options [see (63)]. For replies to some of these criticisms, see Robinson and Berridge (64); Robinson et al. (65).
